# Vasoreactivity as a Measure of Kidney Viability During Ex Vivo Normothermic Machine Perfusion

**DOI:** 10.1111/aor.70033

**Published:** 2025-10-13

**Authors:** Isa M. van Tricht, Baran Ogurlu, Silke S. M. Wolfswinkel, Henri G. D. Leuvenink, Cyril Moers

**Affiliations:** ^1^ Department of Surgery—Organ Donation and Transplantation, University Medical Center Groningen University of Groningen Groningen the Netherlands

**Keywords:** drugs, kidney, normothermic machine perfusion, pharmacology, porcine, transplantation, vasoreactivity, warm ischemia

## Abstract

**Background:**

Normothermic machine perfusion (NMP) could serve as a platform to assess deceased‐donor kidney viability before transplantation, yet it remains unclear which parameters indicate renal viability. As vascular integrity is important for adequate renal function after transplantation, this study aimed to investigate the influence of warm ischemic injury on vascular smooth muscle cell (VSMC) responsiveness to vasoactive drugs during NMP.

**Methods:**

Fourteen porcine kidneys (*n* = 7 per group) were exposed to either 30 or 60 min of warm ischemia (WI), followed by 3.5 h of cold machine perfusion. After cold perfusion, kidneys underwent 4 h of NMP (37°C). During NMP, vasoactive drugs were sequentially infused into the renal artery at 30‐min intervals, starting with epoprostenol (10 μg), followed by dopamine (1 mg), sodium nitroprusside (2 mg), acetylcholine (1 mg), norepinephrine (10 μg), and finally verapamil (2.5 mg).

**Results:**

Renal blood flow during NMP changed significantly in both groups after administration of dopamine, acetylcholine, norepinephrine, and verapamil, but not following epoprostenol and sodium nitroprusside infusion. In kidneys subjected to 30 min of WI, the response to dopamine and norepinephrine was more pronounced, and oxygen consumption and blood pH were higher compared to kidneys that sustained 60 min of WI.

**Conclusion:**

This study indicates that prolonged WI damage diminishes the contractility of VSMCs through the α‐adrenergic receptors. Our findings suggest that the renal vascular responses to dopamine and norepinephrine, as well as decreased oxygen consumption and blood pH, could serve as objective indicators to quantify warm ischemic injury during renal NMP.

## Graphical abstract

This study aimed to investigate the influence of warm ischemic injury on renal vascular responsiveness to vasoactive drugs during normothermic machine perfusion (NMP). We found that the vascular responses to dopamine and norepinephrine could be used as objective indicators to assess kidney viability during NMP.

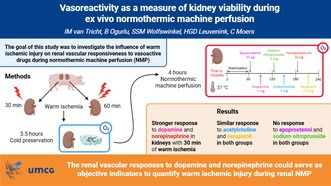

## 1 Introduction

Normothermic machine perfusion (NMP) is a near‐physiological preservation technique during which a warm (37°C) perfusion solution is circulated through a donor kidney. This allows cellular metabolism to resume and could permit organ viability assessment prior to transplantation [1]. Enabling the assessment of renal viability is essential to ensure sufficient quality, as organs of diminished quality, for instance due to extensive warm ischemia (WI), lead to suboptimal outcomes after transplantation [2]. Current ex vivo organ assessment strategies rely on monitoring parameters such as urine production, creatinine clearance, or inulin clearance during NMP [3, 4]. However, the extent to which these in vivo physiology‐derived functional markers accurately reflect kidney viability during ex vivo machine perfusion remains uncertain [5]. Previous research suggests that renal viability assessment might be conducted more reliably through the observation of responses of the kidney to external stimuli during NMP [6, 7]. As vasomotor integrity is important for proper renal functioning [8], exploring the effectiveness of various pathways inducing vasoconstriction or vasodilation in vascular smooth muscle cells (VSMCs) during NMP could provide insights into the susceptibility of VSMCs to warm ischemic injury.

In vivo, the vascular tone in kidneys is moderated by a balance of endogenous vasoconstrictive and vasodilatory substances, which affect the endothelium and the VSMCs. Essentially, contraction or relaxation of VSMCs depends on the activity of the enzymes myosin light‐chain kinase (MLCK) and myosin light‐chain phosphatase (MLCP). MLCK activity is calcium‐dependent and leads to vasoconstriction after the calcium–calmodulin complex is formed. Conversely, vasodilation is induced through MLCP activity when VSMCs are depleted of calcium [9]. Important endogenous vasodilators produced by the endothelium are prostacyclin and nitric oxide (NO). The formation of cyclic adenosine monophosphate (cAMP) and cyclic guanosine monophosphate (cGMP) in VSMCs is promoted by prostacyclin and NO, respectively. Both cAMP and cGMP decrease the calcium concentration in VSMCs through several downstream steps, subsequently favoring vasodilation by preventing the formation of the calcium–calmodulin complex [10, 11, 12, 13]. Epoprostenol, a prostacyclin analog, and sodium nitroprusside (SNP), an NO donor, have both been administered during renal NMP to provide vasodilation [1].

In contrast, vasoconstrictors conventionally induce a rapid influx of calcium into VSMCs through calcium channels, as well as a release of calcium from the intracellular storage, to promote the formation of the calcium–calmodulin complex and subsequent activation of MLCK [14]. Norepinephrine is a well‐known vasoconstrictor that exerts its effects through binding to the α‐adrenergic receptors. Its less potent precursor, dopamine, similarly binds to these receptors, though only at higher doses [15]. Additionally, acetylcholine, commonly known as a vasodilator, exhibits vasoconstrictive effects when bound to muscarinic receptors on VSMCs [16]. As vasoconstriction is dependent on intracellular calcium, depletion of calcium using a calcium channel blocker (CCB), such as verapamil, inhibits MLCK. Similar to epoprostenol and SNP, verapamil has been used during renal NMP for vasodilative purposes [1]. Although the regulation of vascular tone has been well documented in vivo, it is unclear how vasoactive drugs work in an ex vivo setting, and how warm ischemic injury affects either of these pathways.

This preclinical study aimed to investigate the effect of warm ischemic injury on vasodilation and vasoconstriction in kidneys during NMP, through administration of epoprostenol, dopamine, SNP, acetylcholine, norepinephrine, and verapamil.

## 2 Methods

### 2.1 Study Design

**FIGURE 1:**
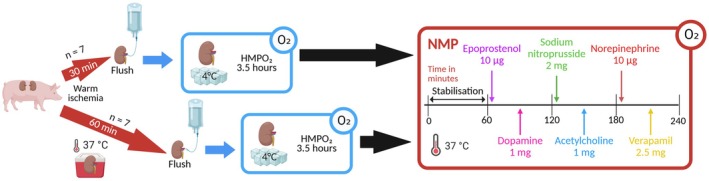
Schematic study design. Kidneys underwent either 30 or 60 min of WI, after which they were flushed and preserved via HMPO_2_ for 3.5 h. Afterwards, kidneys were subjected to a vasoreactivity assessment protocol during 4 h of NMP. HMPO_2_, oxygenated hypothermic machine perfusion; NMP, normothermic machine perfusion; WI, warm ischemia. [Color figure can be viewed at wileyonlinelibrary.com]

A total of 14 kidneys (*n* = 7 per group) were exposed to either 30 or 60 min of WI at 37°C prior to a cold (1°C–4°C) vascular flush with Histidine‐Tryptophan‐Ketoglutarate (HTK) solution, after which they underwent a period of oxygenated hypothermic machine perfusion (HMPO_2_) at 1°C–10°C for 3.5 h. During subsequent normothermic (37°C–38°C) machine perfusion, kidneys were exposed to a vasoreactivity assessment protocol for 4 h. After stabilization in the first hour of NMP, vasoactive drugs were sequentially infused just upstream of the renal artery at 30‐min intervals. Drugs were administered in the following order: 10 μg of epoprostenol; 1 mg of dopamine; 2 mg of SNP; 1 mg of acetylcholine; 10 μg of norepinephrine; 2.5 mg of verapamil (Figure 1).

### 2.2 Kidney Procurement and Preparation

Viable porcine kidneys sourced from female domestic landrace pigs, alongside autologous blood, were acquired from a local abattoir (Kroon Vlees, Groningen, the Netherlands) as previously described by our group [7, 17, 18]. As kidneys are considered waste material from the slaughterhouse, no animal ethics committee approval was required. Prior to procurement, pigs underwent stunning via bi‐temporal electric shock, followed by standard exsanguination procedures typical to slaughterhouse operations. Autologous blood collection was done in a beaker containing 25,000 units of heparin (LEO pharma, Ballerup, Denmark). Kidneys underwent 30 min of WI at 37°C during their procurement from each slaughterhouse pig. Upon retrieval, kidneys were surgically dissected, exposing the vessels and removing perirenal fat. Next, the renal artery was cannulated utilizing an in‐house designed cannula, and the ureter was cannulated with an 8 Ch nasogastric feeding tube (Nutrifit Health Ltd., Caterham, UK). Kidneys allocated to the 60‐min WI group were sealed in a plastic bag and subsequently immersed in a plastic container containing water at 37°C to inflict additional WI until the initiation of cold vascular flush. Following the designated duration of WI, kidneys underwent a cold flush out using 450 mL of HTK (Custodiol Köhler, Dr. Franz Köhler Chemie GmbH, Bensheim, Germany) at 1°C–4°C, with a hydrostatic pressure of 100 cmH_2_O. Subsequently, kidneys were connected to an HMPO_2_ device and perfused with University of Wisconsin machine perfusion solution (Belzer UW‐MP solution, Bridge to Life Ltd., Columbia, SC, USA) for 3.5 h, maintaining a sinusoidal arterial pressure of 30/20 mmHg at 60 beats per minute (BPM). To prepare red blood cells (RBCs) for perfusion, autologous whole blood was depleted of leukocytes using a leukocyte filter (BioR 02 plus BS PF, Fresenius Kabi, Zeist, the Netherlands), followed by centrifugation (at 20°C for 20 min at 2000G, radius 184). Subsequently, supernatant blood plasma and platelets were removed. The remaining RBCs underwent one washing step with phosphate‐buffered saline (PBS), followed by repeated centrifugation at the same settings. After this washing step, the supernatant layer of PBS was removed, and 168 mL of almost‐pure autologous RBCs were isolated for each NMP procedure.

### 2.3 Normothermic Machine Perfusion

The perfusion circuit, as previously described by our group [7, 17, 18], consisted of a pump unit and a centrifugal pump (Medos DeltaStream DP2, Medos Medizintechnik AG), an oxygenator (Hilite 800 LT, Medos Medizintechnik AG), a LifePort organ chamber (Organ Recovery Systems), and a water bath. Perfusion pressure was measured at the distal end of the cannula (TruWave disposable pressure transducer, Edwards Lifesciences, Irvine, CA, USA). A counterflow of 3 mL/h with sodium chloride 0.9% (Fresenius Kabi) was provided over the pressure line. Flow was monitored with an ultrasonic flow sensor (CG Series Ultrasonic Clamp‐on Flow Sensor, CG‐038, XY‐TEK, Shanghai, China), and temperature was measured directly underneath the kidney (Heraeus Nexensos PT100 Surface Sensor (W‐SZK), Heraeus Nexensos GmbH, Kleinostheim, Germany). The circuits were primed with NMP perfusate (Table 1), and oxygenated with carbogen (95% O_2_ / 5% CO_2_) at a gas flow rate of 0.5 L/min. Kidneys were reperfused at 37°C (NMP) with a sinusoidal arterial pressure of 110/70 mmHg at 60 BPM. Arterial pressure, perfusate flow, and renal temperature were measured continuously throughout the warm perfusion. Glucose and pH levels were not actively corrected during NMP.

**TABLE 1:** Perfusate composition.

Component	Amount	
Autologous red blood cells	168	mL
Aminoplasmal 10% (B. Braun Melsungen AG, Melsungen, Germany)	10	mL
Calcium gluconate 10% (B. Braun)	6	mL
Cernevit (Baxter) dissolved in 5 mL sodium chloride 0.9% (Fresenius Kabi Nederland B.V., Zeist, the Netherlands)	0.5	mL
Glucose 5% (Baxter B.V., Utrecht, the Netherlands)	14	mL
Heparin 5000 IU/mL (LEO pharma, Ballerup, Denmark)	0.25	mL
Magnesium sulphate 100 mg/mL (Teva Nederland B.V., Haarlem, the Netherlands)	0.7	mL
Mannitol 15% (Baxter)	5	mL
Potassium chloride 1 mmol/L (Centrafarm B.V., Etten‐Leur, the Netherlands)	0.5	mL
Sterile water (Fresenius Kabi)	155	mL
Sodium bicarbonate 8.4% (B. Braun)	18	mL
Sodium chloride 0.9% (Fresenius Kabi)	355	mL
Sodium phosphate 3 mmol/mL (Apotheek A15, Gorinchem, the Netherlands)	0.15	mL
Bovine Serum Albumin standard grade (Capricorn Scientific, Ebsdorfergrund, Germany)	20	gr
Cefazolin (Fresenius Kabi)	1	gr
Creatinine (Sigma‐Aldrich, Zwijndrecht, the Netherlands)	57.6	mg

### 2.4 Dose Determination and Drug Preparation

The doses for epoprostenol (Flolan, GlaxoSmithKline, Brentfort, United Kingdom), SNP (sodium nitroprusside dihydrate, 71 778 Sigma), and verapamil (2.5 mg/mL, Centrafarm B.V., Breda, The Netherlands) for this experiment were derived from doses that have been used in previous NMP research [1]. As epoprostenol has been provided at 10 μg [19], and verapamil has been used as a starting bolus of 2.5 mg [20], these doses were used in this research. Moreover, SNP has been infused at 25 mg/h [21]. To aim at potential vasodilation for at least 5 min, a bolus of 2 mg SNP was calculated to be required.

Three pilots were performed to determine the doses for dopamine (dopamine hydrochloride 40 mg/mL, Hikma Pharmaceuticals, Terrugem, Portugal), acetylcholine (acetylcholine chloride, A6625 Sigma), and norepinephrine (1 mg/mL, Centrafarm B.V., Breda, The Netherlands) using kidneys subjected to 30 min of WI and treated according to the experimental protocol described above. These pilots were intended solely for dose selection and were not designed for quantitative or statistical analysis. After initiating NMP and a stabilization period of 60 min, dose–response curves were derived for each drug, starting with doses of 1 ng. The renal blood flow (RBF) was observed after each bolus administration, and the doses were increased stepwise by at least a 10‐fold if no alteration in RBF could be registered within 5 min. A constant and reproducible deviation from the baseline RBF was repeatedly observed at a dose of 1 mg for dopamine, at 1 mg for acetylcholine, and at 10 μg for norepinephrine, whereas doses lower than the final selected amounts produced no discernible change. Hence, these were the final doses for the experiments.

To prepare the drugs for the experiments, epoprostenol was first dissolved in the corresponding solvent, and acetylcholine and SNP were dissolved in sodium chloride 0.9% (Fresenius Kabi). All drugs were further diluted with sodium chloride 0.9% (Fresenius Kabi) until the final doses were reached and were subsequently dissolved in sodium chloride 0.9% (Fresenius Kabi) to achieve a final volume of 5 mL. Each bolus was administered in approximately 10 s just upstream of the renal artery at 30‐min intervals.

### 2.5 Perfusate and Urine Measurements

Perfusate samples, and arterial and venous blood gas samples were taken 10 min prior to NMP and after 15 min of NMP. Urine was collected for 15 min at the end of every hour and recirculated after sampling [22]. Venous and urine samples, as well as arterial and venous blood gas samples (ABL90 FLEX; Radiometer, Zoetermeer, The Netherlands) were taken hourly. Perfusate and urine concentrations of creatinine and sodium, and cellular injury markers lactate dehydrogenase (LDH) and aspartate aminotransferase (ASAT) in the perfusate were determined at the clinical laboratory of the University Medical Center Groningen using routine clinical assays. Hyaluronan (HA) was measured in venous samples using a commercial kit (Hyaluronan DuoSet ELISA DY3614, R&D Systems Inc., Minneapolis, MN) according to the manufacturer's protocol. Creatinine clearance, fractional excretion of sodium, and oxygen consumption were calculated as previously described (Supporting Information) [17].

### 2.6 Statistical Analyses

Statistical analyses and data visualization were performed with R version 4.3.3 (R Foundation for Statistical Computing, Vienna, Austria). Normality of the data was assessed with the Shapiro–Wilk test and Q‐Q plots. Continuous data with a normal distribution were analyzed using analysis of variance and Tukey's multiple comparison test for differences in means per time point. The Kruskal‐Wallis test with Dunn's multiple comparison test was used to compare mean ranks per time point for non‐normally distributed continuous data. RBF data was normally distributed, and a paired t‐test was used to compare RBF before and after drug administration within groups. For short‐acting drugs, the maximum VSMC response, expressed as a maximum increase or decrease in RBF, was calculated as a mean of 10 s. For the long‐acting drug verapamil, the maximum response was calculated as the mean from the maximum increase in RBF. A Student's *t*‐test was used to compare RBF values between groups. A two‐sided *p* ≤ 0.05 was considered to indicate statistical significance.

## 3 Results

Fourteen porcine kidneys (*n* = 7 per group) were included. There were no significant differences in renal weight and baseline blood gas values prior to NMP, as well as urine production and renal weight gain during NMP (Table 2).

**TABLE 2:** Kidney characteristics before and after NMP, important baseline perfusate blood gas values, and urine production during NMP.

	30‐min WI group	60‐min WI group	*p*
Kidney characteristics (Mean ± SD)			
Warm ischemia time (minutes)	30.1 ± 0.38	60 ± 0	< 0.0001
Kidney weight before NMP (grams)	280.86 ± 36.09	290.14 ± 25.04	0.59
Kidney weight after NMP (grams)	446.86 ± 63.86	462.71 ± 44.21	0.60
Percentual weight gain during NMP (%)	60.0 ± 20.4	59.7 ± 11.6	0.97
Baseline blood gas values (Mean ± SD)			
Perfusate pH	7.52 ± 0.02	7.51 ± 0.02	0.24
Lactate	0	0	1
Urine (Median ± IQR)			
Total production during NMP (mL/100 g)	68.54 ± 105.99	64.33 ± 82.64	0.80
Median production per hour during NMP (mL/h/100 g)	18.42 ± 33.25	13.88 ± 17.49	0.49

### 3.1 Vascular Parameters

**FIGURE 2:**
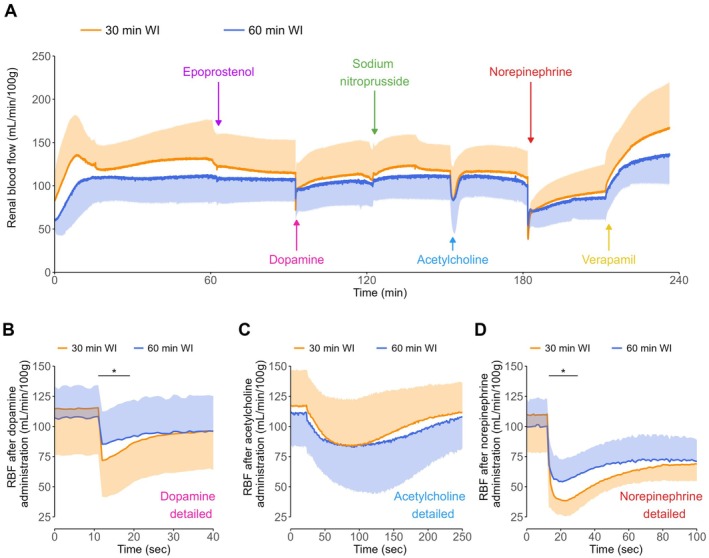
Renal blood flow (A), and changes in RBF after dopamine (B), acetylcholine (C), and norepinephrine (D) administration during normothermic machine perfusion. Means and standard deviations are shown. RBF, renal blood flow; WI, warm ischemia. *p < 0.05. [Color figure can be viewed at wileyonlinelibrary.com]

Overall RBF, including RBF before administration of each drug, did not significantly differ between the 30‐min and the 60‐min WI groups during NMP (Figure 2A). Additionally, RBF did not significantly change after administration of epoprostenol and SNP in both groups.

Dopamine administration resulted in an immediate significant reduction in RBF of 30.4% ± 11.6% in the 30‐min WI group (from 115 ± 38 to 81 ± 30 mL/min/100 g, *p* < 0.001) and 17.6% ± 10.2% in the 60‐min WI group (from 107 ± 25 to 90 ± 28 mL/min/100 g, *p* < 0.001). Additionally, the reduction in RBF was significantly stronger in the 30‐min WI group compared to the 60‐min WI group (*p* < 0.05) (Figure 2B).

In contrast, though the administration of acetylcholine resulted in a gradual decrease in RBF of 29.9% ± 24.3% in the 30‐min WI group (from 117 ± 30 to 82 ± 41 mL/min/100 g, *p* < 0.05) and 28.2% ± 26.4% in the 60‐min WI group (from 111 ± 27 to 81 ± 38 mL/min/100 g, *p* < 0.05), the extent of the responses was comparable between groups (Figure 2C). RBF was not significantly altered at the end of the 30‐min interval compared to RBF before administration of both dopamine and acetylcholine.

Similar to dopamine, RBF decreased significantly immediately after norepinephrine administration with 64.9% ± 10.3% in the 30‐min WI group (from 115 ± 30 to 40 ± 13 mL/min/100 g, *p* < 0.001) and with 51.3% ± 12.5% in the 60‐min WI group (from 108 ± 24 to 53 ± 16 mL/min/100 g, *p* < 0.001). This reduction was significantly stronger in the 30‐min WI group compared to the 60‐min WI group (*p* < 0.05) (Figure 2D).

Administration of verapamil resulted in a significant gradual increase in RBF of 79.7% ± 31.9% in the 30‐min WI group (from 93 ± 31 to 164 ± 51 mL/min/100 g, *p* < 0.001) and with 60.1% ± 24.3% in the 60‐min WI group (from 86 ± 25 to 135 ± 32 mL/min/100 g, *p* < 0.001), but the magnitude of these responses did not differ significantly between both groups.

As norepinephrine provided a constrictive vascular tone, the response to verapamil was quantified by comparing RBF after verapamil administration with RBF before adding norepinephrine, to evaluate the potential of verapamil to induce vasodilation without prior constriction. RBF would have increased by 41.0% ± 17.1% (from 115 ± 30 to 164 ± 51 mL/min/100 g, *p* < 0.001) in the 30‐min WI group and by 25.0% ± 15.8% (from 108 ± 24 to 135 ± 32 mL/min/100 g, *p* < 0.001) in the 60‐min WI group, and this would have been comparable in both groups.

### 3.2 Functional Parameters

**FIGURE 3:**
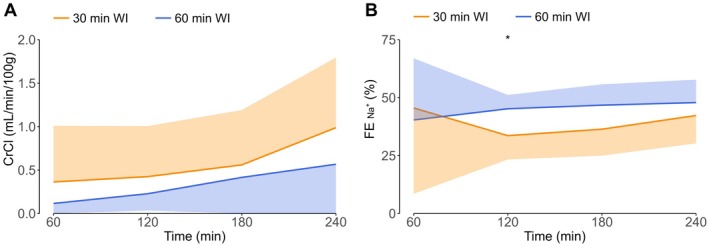
Creatinine clearance (A) and fractional sodium excretion (B) during normothermic machine perfusion. Medians and interquartile ranges are shown for CrCl; means and standard deviations are shown for FE_Na_^+^. CrCl, creatinine clearance; FE_Na_^+^, fractional sodium excretion; WI, warm ischemia. *p < 0.05. [Color figure can be viewed at wileyonlinelibrary.com]

Creatinine clearance was calculated as an indication of glomerular filtration rate (Figure 3A). Creatinine clearance during NMP was very low in both groups, as is common for a porcine kidney in an ex vivo setting [5, 18]. Throughout NMP, creatinine clearance was stable in the 30‐min WI group (0.5 ± 0.6 mL/min/100 g). For the 60‐min WI group, creatinine clearance increased significantly from 0.1 ± 0.1 mL/min/100 g in the first hour of NMP to 0.6 ± 1.2 mL/min/100 g in the last hour of NMP (*p* < 0.01) (statistical significance not indicated in Figure 3A), but did not differ from the 30‐min WI group at any time point.

Fractional sodium excretion (FE_Na_^+^) was calculated to evaluate tubular transport function during NMP (Figure 3B). Similar to creatinine clearance, FE_Na_^+^ remained relatively stable throughout NMP in the 30‐min WI group (39.5% ± 17.7%) and the 60‐min WI group (45.1% ± 12.9%). At the second hour of NMP, the FE_Na_^+^ was significantly lower in the 30‐min WI group (33.6% ± 10.3%) compared to the 60‐min WI group (45.2% ± 6.0%, *p* < 0.05), but was comparable at all other time points.

### 3.3 Metabolic Parameters

**FIGURE 4:**
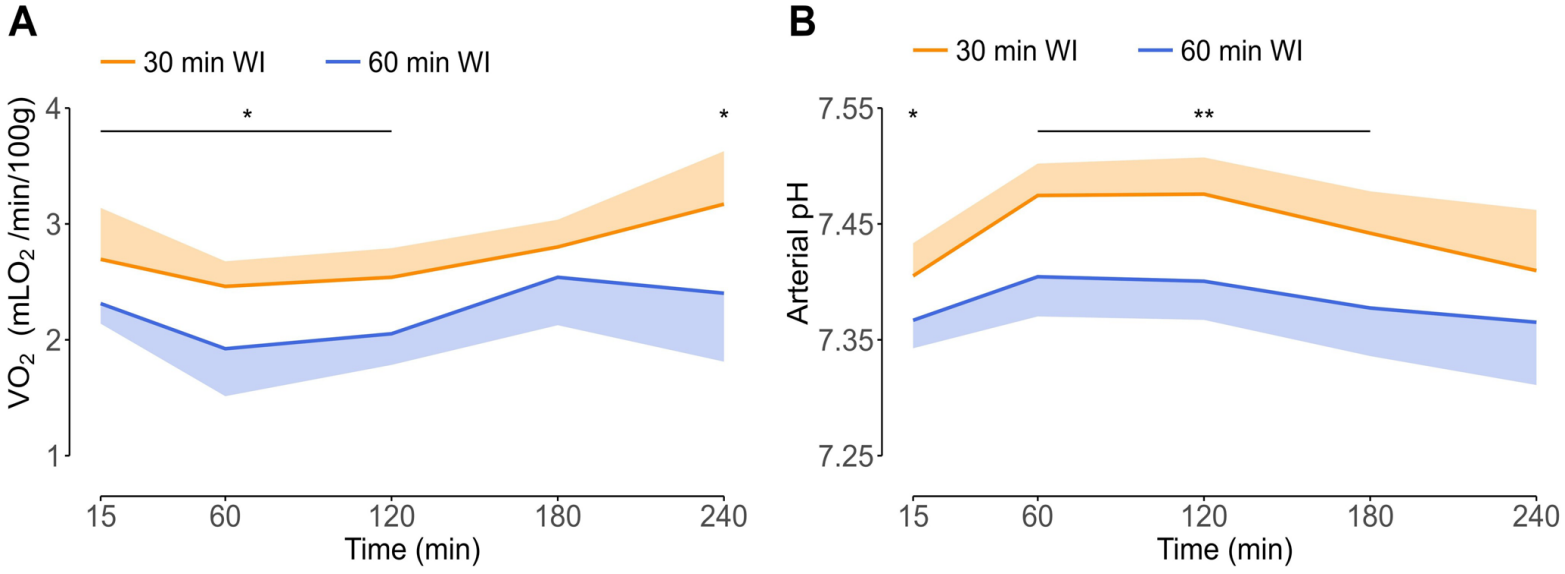
Oxygen consumption (A) and arterial pH (B) during normothermic machine perfusion. Medians and interquartile ranges are shown for VO_2_; means and standard deviations are shown for arterial pH. VO_2_, Oxygen consumption; WI, warm ischemia. *p < 0.05, **p < 0.01. [Color figure can be viewed at wileyonlinelibrary.com]

Oxygen consumption (VO_2_) was calculated to evaluate renal cellular metabolism during NMP (Figure 4A). VO_2_ in the 30‐min WI group gradually increased from 2.5 ± 0.2 mLO_2_/min/100 g in the first hour to 3.3 ± 0.5 mLO_2_/min/100 g in the last hour of NMP (*p* < 0.05) (statistical significance not indicated in Figure 4A). Additionally, VO_2_ in the 60‐min WI group (2.3 ± 0.4 mLO_2_/min/100 g) was significantly lower compared to the 30‐min WI group (2.7 ± 0.3 mLO_2_/min/100 g) at all time points, except for the third hour during NMP (*p* < 0.05).

The arterial pH was measured via blood gas analyses during NMP (Figure 4B). For the 30‐min WI group, the pH increased significantly from 7.41 ± 0.03 at 15 min to 7.47 ± 0.03 after the first hour (*p* < 0.01), and then gradually decreased to 7.41 ± 0.05 at the end of NMP (*p* < 0.05) (statistical significance not indicated in Figure 4B). Throughout NMP, the arterial pH was significantly higher in the 30‐min WI group compared to the 60‐min WI group (7.44 ± 0.03 vs. 7.38 ± 0.03, *p* < 0.05) but became comparable at the end of NMP. This difference remained visible in the venous pH, which was also higher in the 30‐min WI group compared to the 60‐min WI group (7.41 ± 0.04 v. 7.35 ± 0.04, *p* < 0.05).

Lactate levels were measured via blood gas analyses as well. For the 30‐min WI group, lactate increased significantly from 1.89 ± 0.72 mmol/L at 15 min to 4.03 ± 1.82 mmol/L at the end of NMP (*p* < 0.01), and for the 60‐min WI group from 2.33 ± 0.40 mmol/L at 15 min to 4.01 ± 1.29 mmol/L at the end of NMP (*p* < 0.05). However, at no point did the lactate levels differ significantly between both groups.

### 3.4 Renal Injury

**FIGURE 5:**
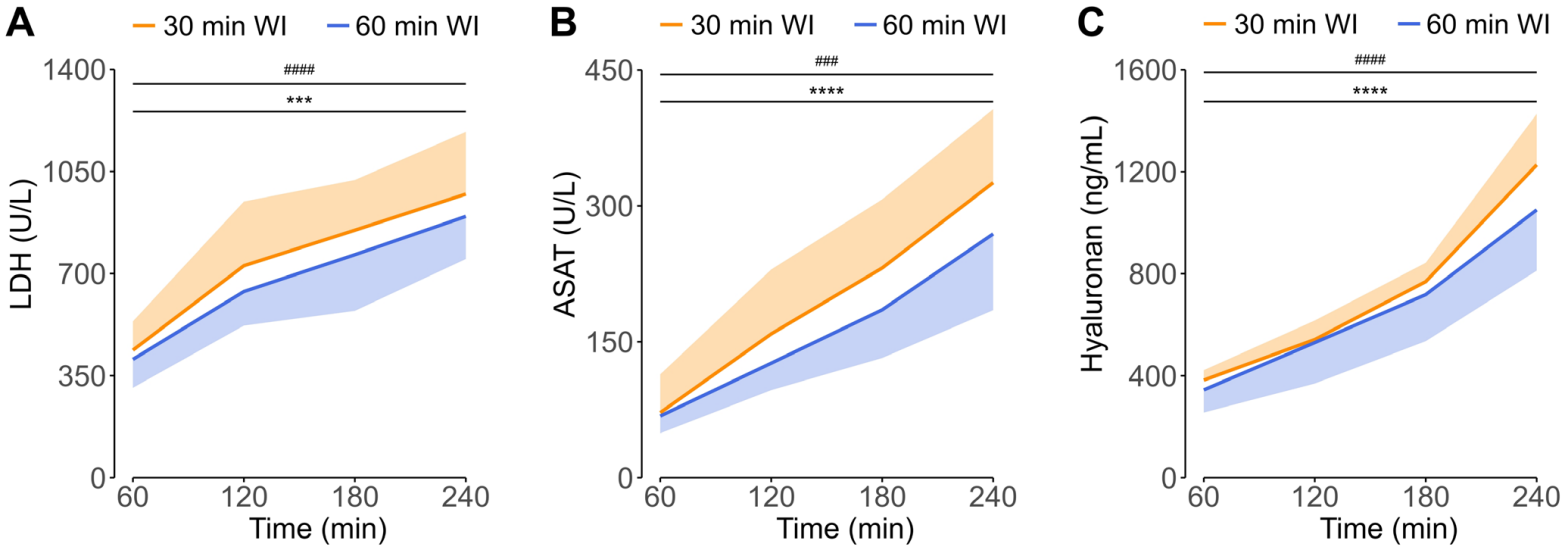
Levels of lactate dehydrogenase (A), aspartate aminotransferase (B), and hyaluronan (C) during normothermic machine perfusion. Means and standard deviations are shown. ASAT, aspartate aminotransferase; LDH, lactate dehydrogenase; WI, warm ischemia. Increase during NMP for 30‐min WI group, ***p < 0.001, ****p < 0.0001. Increase during NMP for 60‐min WI group, ###*p* < 0.001, ####*p* < 0.0001. [Color figure can be viewed at wileyonlinelibrary.com]

Levels of LDH and ASAT during NMP were measured as indicators of cellular injury. The levels of LDH were not significantly different between the two groups, but did increase significantly from 437.43 ± 98.49 U/L in the first hour to 972.43 ± 213.07 U/L at the end of NMP (*p* < 0.001) in the 30‐min WI group, and from 405.86 ± 97.18 U/L to 895.86 ± 145.91 U/L (*p* < 0.0001) in the 60‐min WI group (Figure 5A). Similar to LDH, ASAT levels increased during NMP from 71.86 ± 42.20 U/L to 325.57 ± 81.02 U/L (*p* < 0.0001) in the 30‐min WI group and from 68.14 ± 18.93 U/L to 269.00 ± 84.02 U/L (*p* < 0.001) in the 60‐min WI group, but did not significantly differ between both groups (Figure 5B). To assess the extent of endothelial glycocalyx injury, perfusate hyaluronan (HA) was measured (Figure 5C). The levels of HA did not differ significantly between the groups, but in line with levels of LDH and ASAT, HA did increase significantly during NMP from 382.86 ± 38.89 ng/mL to 1226.71 ± 199.67 ng/mL (*p* < 0.0001) in the 30‐min WI group and from 344.57 ± 88.56 ng/mL to 1050.14 ± 238.00 ng/mL (*p* < 0.0001) in the 60‐min WI group.

## 4 Discussion

To the best of our knowledge, this is the first study to administer various vasoconstrictive and vasodilative drugs during renal NMP in order to evaluate the vascular response in porcine kidneys exposed to either 30 or 60 min of WI. Our data show that prolonged exposure to WI results in a diminished, but not absent, response to dopamine and norepinephrine when compared to less injured kidneys. Both groups exhibited similar responses to verapamil and acetylcholine while remaining unresponsive to epoprostenol and SNP. Additionally, we found that renal oxygen consumption and arterial and venous pH during NMP were higher in kidneys subjected to 30 min of WI compared to those exposed to 60 min. Nevertheless, the selected cellular and vascular injury markers were not significantly different in kidneys subjected to prolonged ischemic injury compared to healthier kidneys.

The clear decrease in RBF after dopamine, acetylcholine, and norepinephrine administration in both groups confirms the integrity of VSMCs and the functionality of calcium‐dependent vasoconstrictive pathways, as well as MLCK activity in kidneys in our study. Additionally, the recovery of RBF after dopamine and acetylcholine administration suggests adequate MLCP activity to reverse the constrictive vascular tone. In line with the findings of Bath et al. [6], we observed no difference in maximum vasoconstriction after acetylcholine administration between both groups. Interestingly, despite a similarity in the extent of vasoconstriction via the muscarinic receptor, vasoconstriction through the α‐adrenergic receptor—induced by both dopamine and norepinephrine—was more pronounced in kidneys exposed to 30 min of WI. Therefore, our findings suggest that warm ischemic injury does not affect vascular affinity to α‐adrenergic receptors, but likely diminishes the efficacy of dopamine and norepinephrine in VSMCs [23]. Similar alterations in the effectiveness of comparable drugs after ischemia have been reported in cardiac tissue studies [24, 25], but the underlying mechanisms behind this decreased efficacy remain unclear. Nevertheless, our results suggest that WI selectively affects the α‐adrenergic receptors and not the muscarinic receptors in kidneys. This notion could allow objective quantification of warm ischemic injury through dopamine and norepinephrine administration.

Although epoprostenol and SNP have been previously used as vasodilators in perfusion protocols [1], their effectiveness was not observed in our study. Both drugs are short‐acting and were provided at supraphysiological doses; however, the anticipated increase in RBF remained absent. It could be possible that these drugs require continuous infusion to sufficiently activate their downstream pathways that enable vasodilation. Nevertheless, prior research confirmed that ischemic artery rings were less sensitive to SNP [26]. As NO is an important mediator of endothelial dysfunction and vasoconstriction after ischemic injury [27], a disruption of the NO pathway due to sustained WI could explain the lack of vasodilatory response to SNP in kidneys from our study. Additionally, evidence on the effects of prostacyclin infusion on RBF is conflicting, with either vasoconstriction, vasodilation, or unchanged vascular tone being reported depending on the dose [28, 29, 30]. Regardless, kidneys in this study exhibited no vasodilation through either the cAMP or cGMP pathway, suggesting that warm ischemic injury impairs these vasodilative mechanisms. Therefore, epoprostenol and SNP should be used cautiously in porcine renal NMP if the intended goal is vasodilation, as their efficacy should be confirmed first.

While calcium‐independent pathways failed to induce vasodilation, the renal response to verapamil was pronounced, aligning with findings from liver NMP [31]. This long‐acting CCB induced a gradual yet considerable increase in RBF and, consistent with previous findings [32], reversed the preceding vasoconstrictive tone. Moreover, verapamil administration would likely have resulted in increased RBF without the vasoconstrictive tone prior to norepinephrine administration, showcasing its potential as a vasodilator. These findings suggest that L‐type calcium channels and calcium‐dependent vasodilative mechanisms remain functional in kidneys, irrespective of their exposure to WI. This is further supported by the rapid decrease in RBF following the administration of the calcium‐dependent vasoconstrictors dopamine, norepinephrine, and acetylcholine. Additionally, the marked increase in RBF after verapamil administration supports the use of CCBs to induce vasodilation during NMP.

Creatinine clearance and FE_Na_^+^ are often reported as indicators of renal function. In this study, we observed no significant differences between groups in these functional parameters. Both Hamelink et al. [5] and Hosgood et al. [33] found that kidneys exposed to minimal or 15 min of WI exhibited significantly lower FE_Na_^+^ and higher creatinine clearance during NMP compared to kidneys subjected to 75 min or 90 and 120 min of WI, respectively. This suggests that prolonged WI does affect functional parameters measured during NMP. However, in the same study by Hosgood et al. no differences in FE_Na_^+^ or creatinine clearance were found between kidneys exposed to 15 versus 60 min of WI. Hence, it is no surprise that we found no differences between our experimental groups of 30 and 60 min of WI.

We observed that kidneys subjected to less warm ischemic damage had a higher oxygen consumption and blood pH compared to those with more extensive ischemic damage. As the proximal tubules play a crucial role in acid–base balance and are vulnerable to WI [34], the acidotic profile of kidneys with greater warm ischemic injury is likely attributable to proximal tubule injury. While WI causes mitochondrial dysfunction and affects cellular metabolism [35], its impact on ex vivo oxygen consumption remains unclear. For instance, Hamelink et al. [5] only found an increased oxygen consumption in kidneys with minimal WI injury compared to kidneys subjected to 75 min of WI at the beginning of NMP, but oxygen consumption became comparable between groups thereafter. Similarly, Hosgood et al. [33] did not find differences in oxygen consumption during renal NMP, regardless of the extent of warm ischemic damage. Additionally, the Bohr effect may have contributed to our findings, as the decreased oxygen consumption could be linked to the reduced affinity of hemoglobin for oxygen in the context of a lower pH [36].

Despite clear differences in some parameters between our experimental groups, the levels of injury markers—LDH, ASAT, and hyaluronan—remained similar. These injury markers might not have been specific enough to detect warm ischemic injury. Moreover, several studies have demonstrated that functional parameters and injury markers are influenced by different NMP protocols [18, 37]. Therefore, both functional parameters and injury markers during renal NMP should be interpreted carefully.

This study had several limitations. Firstly, the controllability of the warm ischemia times in a slaughterhouse model could be prone to subtle variability. Additionally, our model did not include reperfusion, and it remains to be investigated whether our viability assessment observations would result in improved outcomes after transplantation. In our study, the drugs were administered in a fixed sequence, which raises the possibility that their effects might have differed at other time points or in a different order during NMP. For instance, vasodilative effects might have been impaired by prior vasoconstriction, or vice versa. Moreover, each drug was administered in a single dose, and although these were determined during multiple pilots, other doses or continuous administration could have elicited different responses. Additionally, drug interference cannot be completely excluded, despite their short‐acting nature and the ability of the kidney to rapidly metabolize them [38, 39, 40]. Furthermore, the study design rendered continuous vasodilation during perfusion impractical due to likely interference with other vasoactive drugs that were to be studied. As vasodilation is usually provided as a standard procedure during NMP, compatible vasodilators should be selected to allow VSMC assessment with dopamine or norepinephrine. In addition, as the influence of the perfusate on certain parameters measured during NMP is well established [18, 37], our findings regarding the differences in pH and oxygen consumption between groups might be attributed to our specific protocol.

In conclusion, this study suggests that the renal vascular response to dopamine and norepinephrine during NMP could serve as a tool to objectively quantify the impact of WI on donor kidneys. In addition, blood pH and oxygen consumption during renal NMP could provide further insights into the extent of warm ischemic damage. Nevertheless, little evidence is available on the association between these parameters and kidney function after transplantation. Therefore, future studies should investigate how blood pH, oxygen consumption, and the vascular response to dopamine and norepinephrine could support the viability assessment of kidneys during NMP. Most importantly, it should be established whether organ selection based on such pre‐transplant viability assessment contributes to improved post‐transplant outcomes.

## Author Contributions

I.M.V.T., B.O., H.G.D.L., and C.M. designed the study. I.M.V.T., B.O., and S.S.M.W. performed the experiments. I.M.V.T. drafted the manuscript. B.O., S.S.M.W., H.G.D.L., and C.M. performed the critical revision.

## Conflicts of Interest

The authors declare no conflicts of interest.

## Supporting information


**Data S1:** aor70033‐sup‐0001‐supinfo.docx.

## Data Availability

The data that support the findings of this study are available from the corresponding author upon reasonable request.
