# Supplementary material for: Vasoreactivity as a Measure of Kidney Viability During Ex Vivo Normothermic Machine Perfusion
Source: Artif Organs. 2025 Oct 13;50(2):281–90. doi: 10.1111/aor.70033 (PMC12993253; doi:10.1111/aor.70033)
Supplement: Supplementary file 1 — Data S1: aor70033‐sup‐0001‐supinfo.docx. [file AOR-50-281-s001.docx]

**Supplementary Material**

Renal oxygen consumption (VO_2_) was calculated using the following formula:

VO_2_ (mL O_2_) = $\left( \left( \left( Hb arterial\times2.4794 \right)+\left( pO2 arterial\times K \right) \right)-\left( \left( 0.024794\times Hb\times SO2 venous \right)+\left( pO2 venous\times K \right) \right) \right)$

Where Hb is the hemoglobin concentration in mmol/L, pO_2_ is the partial oxygen pressure in kPa, K is the solubility constant of oxygen in water at 37°C and equals 0.0225 (mL O_2_ per kPa), and SO_2_ is the saturation in %.

The fractional excretion of sodium (FE_Na_^+^) was calculated using the following formula:

FE_Na_^+^ (%) = $\frac{urinary \left[ Na \right] \times perfusate \left[ creatinine \right]}{\left( urinary \left[ creatinine \right] \times perfusate \left[ Na \right] \right)}\times100$
